# Neutral Lipids, Glycolipids, and Phospholipids, Isolated from Sandfish (*Arctoscopus japonicus*) Eggs, Exhibit Anti-Inflammatory Activity in LPS-Stimulated RAW264.7 Cells through NF-κB and MAPKs Pathways

**DOI:** 10.3390/md18090480

**Published:** 2020-09-21

**Authors:** Weerawan Rod-in, Chaiwat Monmai, Il-sik Shin, SangGuan You, Woo Jung Park

**Affiliations:** 1 Department of Marine Biotechnology, Gangneung-Wonju National University, Gangneung, Gangwon 25457, Korea; kae_ve@hotmail.com (W.R.-i.); bbuayy@gmail.com (C.M.); shinis@gwnu.ac.kr (I.-s.S.); umyousg@gwnu.ac.kr (S.Y.); 2 East Coast Life Sciences Institute, Gangneung-Wonju National University, Gangneung, Gangwon 25457, Korea

**Keywords:** *Arctoscopus japonicus*, lipid, polyunsaturated fatty acids, anti-inflammatory, NF-κB pathway, MAPK

## Abstract

Total lipids were extracted from sandfish (*Arctoscopus japonicus*), and then they were separated into the following three lipid fractions: neutral lipids, glycolipids, and phospholipids. In this study, we analyzed the lipid fractions of *A. japonicus* eggs and we determined their anti-inflammatory activity in RAW264.7 macrophage cells. In these three lipid-fractions, the main fatty acids were as follows: palmitic acid (16:0), oleic acid (18:1n-9), docosahexaenoic acid (DHA, 22:6n-3), and eicosapentaenoic acid (EPA, 20:5n-3). Among the lipid fractions, phospholipids showed the highest concentration of DHA and EPA (21.70 ± 1.92 and 18.96 ± 1.27, respectively). The three lipid fractions of *A. japonicus* significantly suppressed the production of NO in macrophages. Moreover, they also significantly inhibited the expression of *iNOS*, *COX-2*, *IL-6*, *IL-1β*, and *TNF-α*, in a dose-dependent manner. Furthermore, the lipid fractions of *A. japonicus* suppressed the nuclear translocation of NF-κB p65 subunits in a dose-dependent manner. In addition, they attenuated the activation of MAPKs (p38, ERK1/2, and JNK) phosphorylation in LPS-stimulated RAW264.7 cells. These results indicate that all the lipid fractions of *A. japonicus* exert anti-inflammatory activity by suppressing the activation of NF-κB and MAPK pathways. Therefore, the lipid fractions of *A. japonicus* might be potentially used as anti-inflammatory agents.

## 1. Introduction

Fish eggs contain many nutrients, such as amino acids, lipids, vitamins, and minerals. Lipids perform many important structural and functional roles in cell membranes. Moreover, lipids are a major source of energy during embryonic and early developmental stages of larvae. In addition, lipids are a major source of essential fatty acids, which play a pivotal role in the growth, reproductive functions, and in the maintenance of healthy tissues [1,2]. Based on their chemical characteristics, lipids can be divided in two major classes: neutral lipids (triacylglycerols (TAGs), waxes, and terpenes), and polar lipids (phospholipids, glycolipids, sphingolipids, etc.) [3]. Neutral lipids are also known as reserve lipids, which are used as a major source of energy [4,5]. On the other hand, polar lipids are important components of the cell membrane. Some of them can serve as a source of energy while participating in cell signaling and cell-cell interactions [3,6]. For example, glycolipids are membrane lipids that act as cellular markers, and they also participate in cell adhesion, signal transduction, cell-cell interaction, and recognition [7]. Moreover, phospholipids are essential components for the developing eggs in fish, and they also serve as a source of energy during the embryonic and early stages of larval development [8].

Fish lipids are rich in polyunsaturated fatty acids (PUFAs), which act as precursors of lipid mediators and regulate inflammation and immune responses [9]. In particular, eicosapentaenoic acid (EPA, 20:5n-3) and docosahexaenoic acid (DHA, 22:6n-3) are found to be in high concentrations in various species of fish, such as herring [10,11,12], tuna [13], sea bream [14], beluga [15], rainbow trout [16], salmon [12,17,18], boarfish [12], and teleost [2]. Both EPA and DHA exert beneficial effects on various inflammatory diseases, such as Alzheimer’s disease [19], cardiovascular diseases [20], cancer [21,22], diabetes [23,24], thrombosis [12,17,18], and inflammatory activity [25,26]. A recent research study has reported that when n-3 PUFA binds with polar lipids, it exerts differential bioavailability and biological effects upon consumption in contrast to neutral forms of n-3 PUFA [27]. Similarly, in vivo studies have also found that when n-3 PUFA binds with polar lipids, it becomes more bioavailable and bioactive than n-3 PUFA bound to TAG [28].

Lipopolysaccharide (LPS)-stimulated macrophages release inflammatory mediators, such as nitric oxide (NO) and prostaglandin E_2_ (PGE_2_). These compounds induce the expression of immune regulated enzymes, such as nitric oxide synthases (iNOS) and cyclooxygenase-2 (COX-2). Moreover, they also induce the expression of pro-inflammatory cytokines, such as interleukin-1β (IL-1β), IL-6, and tumor necrosis factor (TNF)-α. These cytokines play a pivotal role in eliciting pathogenic inflammatory responses [29]. However, omega-3 fatty acids can inhibit the product of cytokines in LPS-activated human monocytes and murine RAW264.7 macrophages [30,31,32,33]. In LPS-stimulated macrophages, omega-3 fatty acids block the activity of pro-inflammatory transcription factor, such as nuclear factor κB (NF-κB), [26,31,33] and suppress the activity of mitogen-activated protein kinases (MAPKs) [26,34].

Sandfish (*Arctoscopus japonicus*) is a cold-water fish, which is found in the Northwestern Pacific Ocean and, in the east coast of Korea [35,36]. It has been found that *A. japonicus* eggs exhibit antioxidant activity [37,38] and anti-inflammatory activity of peptides, which are derived from enzymatic hydrolysates on RAW264.7 cells [39,40]. Ishihara and Watanabe [41] reported that Japanese sandfish eggs predominantly contained the following fatty acids: palmitic acid, oleic acid, DHA, and EPA. In our preliminary research study, we found that total lipids of *A. japonicus* eggs had high amounts of PUFAs (52.9% of the total fatty acid content; the predominant compounds were DHA [25.9 ± 0.1%] and EPA [21.2 ± 0.5%]). Furthermore, it was found that total lipids exhibit anti-inflammatory activity on macrophages [42]. However, no previous study has examined the fatty acid profiles of individual lipids, which were isolated from *A. japonicus* eggs by fractional distillation. Moreover, no previous study has determined how these lipid-fractions exert anti-inflammatory activity on macrophages. Therefore, the present study was conducted to determine the fatty acid composition of fractionated lipids, which were isolated from *A. japonicus* eggs. These lipid fractions were composed of neutral lipids, glycolipids, and phospholipids. In this study, we also investigated how these lipid fractions exerted anti-inflammatory activity on LPS-stimulated RAW264.7 macrophages.

## 2. Results

### 2.1. Fatty Acid Analysis of A. japonicus Lipid-Fractions (Neutral Lipids, Glycolipids, and Phospholipids), Which were Isolated from A. japonicus Eggs

Gas chromatography (GC)-flame ionization detection (FID) method was used to determine the composition of fatty acids in lipid fractions, which were isolated from *A. japonicus* eggs. These lipid fractions contained neutral lipids, glycolipids, and phospholipids. Table 1 presents the composition of fatty acids in the lipid fractions derived from *A. japonicus* eggs. In all the lipid fractions, the most abundant saturated fatty acids (SFAs) were palmitic acid (16:0). Its composition in different lipid fractions was as follows: neutral lipids (33.17 ± 0.28), glycolipids (26.30 ± 0.27), and phospholipids (19.01 ± 1.27%). In neutral lipids and glycolipids, monounsaturated fatty acids (MUFAs) were the most abundant form of fatty acids (FAs). The concentration of MUFAs in neutral lipids and glycolipids was found to be 46.80 ± 0.79 and 38.68 ± 0.26%, respectively. In contrast, polyunsaturated fatty acids (PUFAs) were the most abundant FAs in phospholipids. The concentration of PUFAs in phospholipids (46.86 ± 3.40%) was found to be significantly higher than that in neutral lipids (16.20 ± 0.70%) and glycolipids (28.79 ± 0.58%). Among the PUFAs, long chain omega-3 fatty acids, such EPA and DHA, were found in high concentrations in phospholipids, followed by glycolipids and neutral lipids, respectively.

### 2.2. The Cytotoxicity of A. japonicus Lipid-Fractions (Neutral Lipids, Glycolipids, and Phospholipids) against RAW264.7 Cells

**Figure 1:**
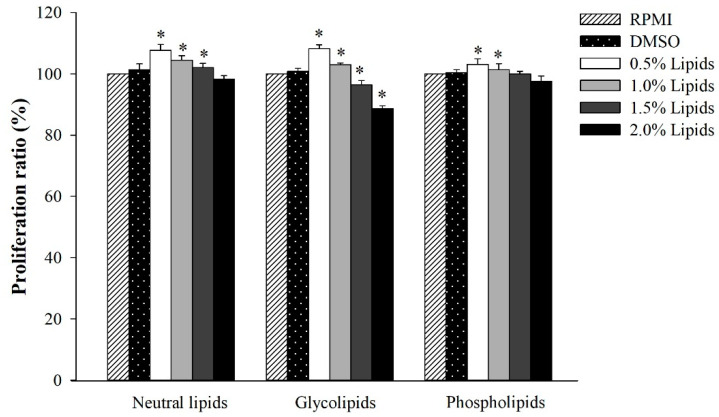
The effect of *A. japonicus* lipid-fractions (neutral lipids, glycolipids and phospholipids) on cell proliferation in RAW264.7 cells. The data were presented as the mean ± SD of three independent experiments. Significant difference was observed at *p* < 0.05 (*) when compared with RPMI medium.

We evaluated the cytotoxic effects of the three fractionated lipids, namely, neutral lipids, glycolipids, and phospholipids, which were derived from *A. japonicus* eggs. For this purpose, we cultured RAW264.7 cells in different concentrations of lipid fractions. Thereafter, the EZ-Cytox Cell Viability Assay Kit was used to measure the proliferation of cells. Figure 1 shows that unlike the negative control, neutral lipids, and phospholipids showed no cytotoxicity up to a concentration of 2.0%. Glycolipids also showed no cytotoxicity when they were present in low concentrations of 0.5% and 1.0% in lipids; however, they showed slight cytotoxicity at higher concentrations of 1.5% and 2%. Nevertheless, the cytotoxicity of glycolipids was slightly lower than that of the negative control.

### 2.3. The Anti-Inflammatory Effect of A. japonicus Lipid-Fractions (Neutral Lipids, Glycolipids, and Phospholipids) against NO Production in LPS-Stimulated RAW264.7 Cells

**Figure 2:**
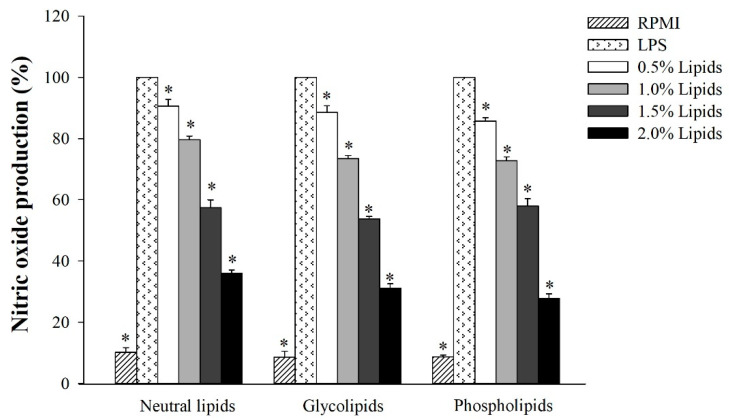
The effect of *A. japonicus* lipid-fractions (neutral lipids, glycolipids and phospholipids) on the production of nitric oxide (NO) in lipopolysaccharide (LPS)-stimulated RAW264.7 cells. The data were presented as mean ± SD of three independent experiments. A significant difference was observed at *p* < 0.05 (*) when compared with LPS.

After isolating the three lipid fractions from *A. japonicus* eggs, we determined their anti-inflammatory activity by measuring the concentration of accumulated nitrite in the culture medium. Griess reagent assay was performed for this purpose. As shown in Figure 2, RAW264.7 cells were treated with various concentrations of each lipid fraction, and the concentration of NO was determined in LPS-stimulated macrophage cells. Our results indicate that neutral lipids, glycolipids, and phospholipids significantly decreased the production of NO in LPS-stimulated RAW264.7 cells in a dose-dependent manner.

### 2.4. The Anti-Inflammatory Effect of A. japonicus Lipid Fractions (Neutral Lipids, Glycolipids, and Phospholipids) against Immune-Associated Gene Expression in LPS-Stimulated RAW264.7 Cells

**Figure 3:**
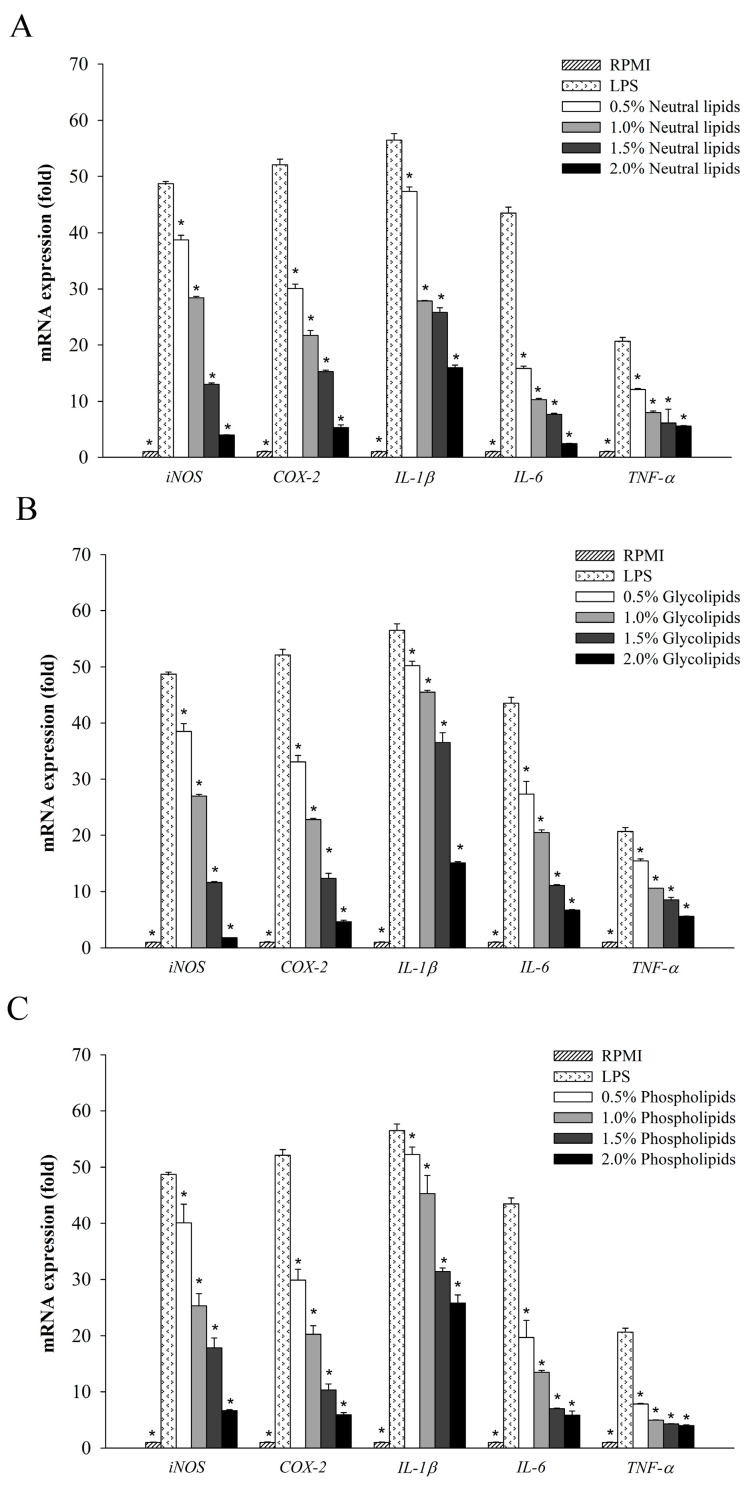
The effect of *A. japonicus* lipid-fractions (neutral lipids, glycolipids and phospholipids) on immune-associated gene expression in LPS-stimulated RAW264.7 cells. (**A**) The relative mRNA expression of neutral lipids; (**B**) The relative mRNA expression of glycolipids; (**C**) The relative mRNA expression of phospholipids. The data were presented as mean ± SD (*n* = 3). A significant difference was observed at *p* < 0.05 when compared with LPS (*).

To investigate whether lipid fractions of *A. japonicus* eggs have anti-inflammatory effects, we analyzed the expression of immune-associated genes by performing real-time quantitative PCR on RAW264.7 cells. Thus, we determined the gene expression level of neutral lipids (Figure 3A), glycolipids (Figure 3B), and phospholipids (Figure 3C). In particular, inflammatory mediator genes, such as *iNOS* and *COX-2*, had significantly decreased expression in the lipid fractions of *A. japonicus* eggs. Moreover, the expression of pro-inflammatory cytokine genes, such as *IL-1β*, *TNF-α*, and *IL-6*, was also inhibited in a dose-dependent manner. However, cell-specific gene expression was slightly and differentially inhibited depending on the concentration of lipid fractions derived from *A. japonicus*.

### 2.5. The Anti-Inflammatory Effects of A. japonicus Lipid Fractions (Neutral Lipids, Glycolipids, and Phospholipids) on the NF-κB and MAPKs Signaling Pathways of LPS-Stimulated RAW264.7 Cells

**Figure 4:**
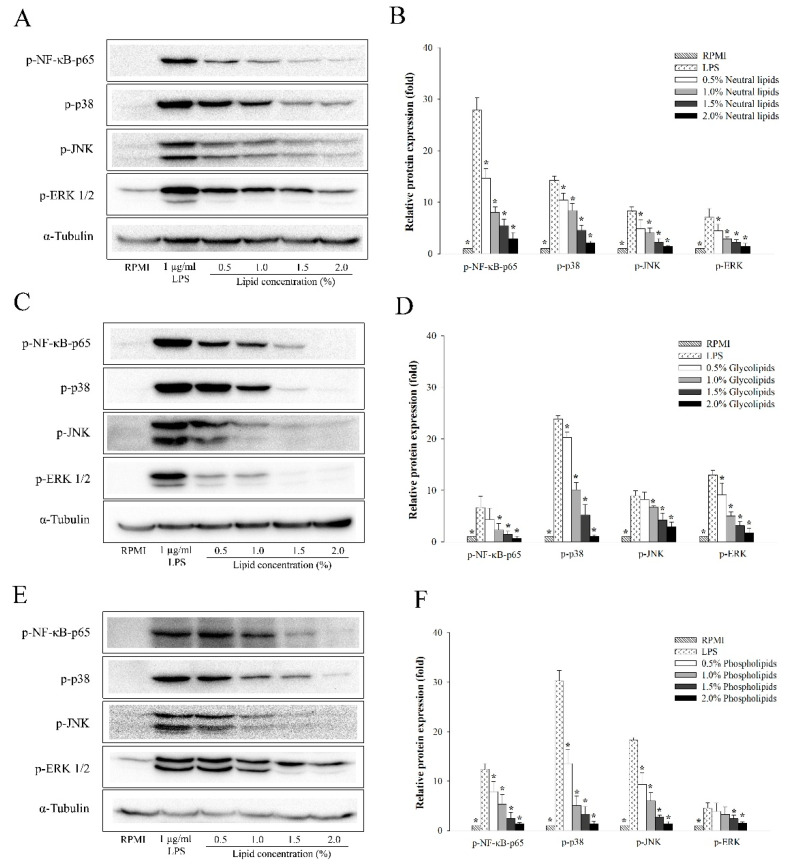
Western blot analysis was performed to determine the effect of lipid fractions (neutral lipids, glycolipids and phospholipids) of *A. japonicus* on the expression of proteins associated with NF-κB and MAPK pathways in LPS-stimulated RAW264.7 cells. (**A**) Western blot analysis of neutral lipids; (**B**) The relative protein expression of neutral lipids; (**C**) Western blot analysis of glycolipids; (**D**) The relative protein expression of glycolipids; (**E**) Western blot analysis of phospholipids; (**F**) The relative protein expression of phospholipids. The data were presented as mean ± SD (*n* = 3). A significant difference was observed at *p* < 0.05 when compared with LPS (*).

To further investigate whether the lipid fractions of *A. japonicus* exhibited anti-inflammatory activity in LPS-stimulated RAW264.7 cells, we determined the protein expression levels of LPS-induced phosphorylation of MAPKs and NF-κB-p65 pathways in RAW264.7 cells by performing western blot analysis. Figure 4 shows that neutral lipids (Figure 4A,B), glycolipids (Figure 4C,D), and phospholipids (Figure 4E,F) suppressed the phosphorylation of NF-κB p65 subunit in a dose-dependent manner, when compared to that in the control.

All the lipid fractions of *A. japonicus* eggs could also inhibit the activation of phosphorylated ERK, JNK, and p38, which are the biomarkers of the MAPK signaling pathway.

## 3. Discussion

Previous studies have shown that EPA (21.2%) and DHA (25.9%) were abundantly present in the total lipids extracted from *A. japonicus* eggs. Moreover, the lipid fractions exhibited anti-inflammatory activity by inhibiting the activity of NF-κB and MAPKs signaling pathways in LPS-stimulated mouse macrophages [42]. In this study, total lipids were extracted from *A. japonicus* eggs and were subjected to fractional distillation. Thus, we obtained the following three lipid fractions: neutral lipids, glycolipids, and phospholipids. Furthermore, we investigated these lipid fractions to determine whether they exhibit anti-inflammatory activity.

The composition of fatty acids in lipid fractions was determined by GC-FID method. Our results indicate that the three lipid fractions, namely, neutral lipids, glycolipids, and phospholipids contained different kinds of fatty acids, such as SFAs, MUFAs, and PUFAs in different ratios (Table 1). In previous literature studies, it has been reported that palmitic acid (16:0) and oleic acid (18:1n-9) were the major fatty acids in herring eggs, and they were further classified as SFAs and MUFAs, respectively. These fatty acids are essential sources of metabolic energy in fish [10]. Likewise, EPA (20:5n-3), DHA (22:6n-3), palmitic acid (16:0), and oleic acid (18:1n-9) were the major constituents of lipid fractions isolated from several fish eggs [11,43,44,45,46]. Although previous studies were based on *Atlantic salmon* and fish roes, their results were similar to those obtained in this study [11,47]. Thus, neutral lipids and glycolipids that were isolated from *A. japonicus* eggs had the highest concentration of MUFAs, while phospholipids were composed of a large amount of PUFAs. In particular, we also observed that EPA and DHA were present in significant amounts in phospholipids. Moreover, EPA and DHA were also predominantly present in other lipid fractions. Interestingly, EPA and DHA are the omega-3 PUFAs that play a pivotal role in eliciting anti-inflammatory responses [48]. The lipid extracts obtained from fish had the highest amount of omega-3 PUFAs, and they exhibited maximum anti-inflammatory activity by stimulating immune cells, such as macrophages. Thus, these lipid extracts were useful in suppressing inflammatory responses [9,48]. Furthermore, it has been found that DHA is more effective than EPA in suppressing LPS-induced production of pro-inflammatory cytokines in macrophages [32]. Marine polar lipids are rich in omega-3 PUFAs, which exhibit strong anti-inflammatory activity and antithrombotic effects on the inflammatory and thrombotic mediators of platelet-activating factor (PAF) and thrombin [12,17,18,49]. Apart from PAF and thrombin, several inflammatory responses are characterized by the release of other critical inflammatory mediators, such as NO and several prostaglandins like PGE_2_.

The inflammatory responses are associated with the release of critical inflammatory mediators, such as NO and PGE_2_. The expression of these mediators is regulated by iNOS and COX-2, respectively, and pro-inflammatory cytokines [50]. Ahmad et al. [51] reported that they extracted lipids from Australian seafood organisms, such as octopus, squid, sardine, salmon, and school prawns. These lipid extracts performed immunomodulatory actions, such as the inhibition of NO and TNF-α production in LPS-stimulated RAW264.7 cells. Similarly, the lipid extracts isolated from blue-green alga (*Nostoc commune*) significantly reduced the expression of *TNF-α*, *IL-1β*, *IL-6*, *COX-2*, and *iNOS* in LPS-stimulated macrophages by inhibiting the activation of NF-κB pathway [52]. Skipjack tuna eyeball oil also exerts anti-inflammatory effects by inhibiting the activity of NF-κB and MAPKs pathways in LPS-induced RAW264.7 cells [53].

Diverse marine organisms exhibit immune biological activities. In this study, we found that the lipid fractions of *A. japonicus* had remarkably suppressed the production of NO in LPS-stimulated macrophage cells (Figure 2). Moreover, the lipid fractions of *A. japonicus* also inhibited the activity of NO by downregulating the expression of *iNOS* and *COX-2*, which are found to be associated with the secretion of NO and PGE_2_. Thus, the lipid fractions of *A. japonicus* suppressed the expression of pro-inflammatory cytokines (Figure 3). This is because pro-inflammatory cytokines such as TNF-α, IL-6, and IL-1β play an important role in eliciting immune responses to a variety of inflammatory stimuli. In this study, the three lipid fractions of *A. japonicus* significantly inhibited the LPS-stimulated mRNA expression of these pro-inflammatory cytokines in a dose-dependent manner (Figure 3). Chen et al. [54] isolated four lipid fractions, namely, total lipids, neutral lipids, glycolipids, and phospholipids from seahorse (*Hippocampus trimaculatus*). They found that these lipid fractions could inhibit the production of NO, IL-6, IL-1β, and TNF-α. The marine neutral lipids, for example, sterols isolated from *Dendronephthya gigantean* exhibited a significant anti-inflammatory activity by inhibiting the production of NO and PGE_2_. Moreover, they also suppressed the expression of pro-inflammatory cytokines, such as *TNF-α*, *IL-1β*, and *IL-6* in LPS-stimulated RAW264.7 macrophages in a dose-dependent manner [55]. Meanwhile, polar lipids that were isolated from marine macroalga also suppressed LPS-induced NO production in macrophage cells by downregulating the expression of *iNOS* [56,57]. Furthermore, glycolipids suppressed LPS-induced vascular inflammation by attenuating the NF-κB pathway and by stimulating the production of NO in endothelial cells [58].

It is well-known that LPS stimulation causes inflammation through several intracellular signaling pathways, such as NF-κB and MAPK [29]. Moreover, NF-κB is an important transcription factor that regulates the expression of immune-related cytotoxic factors, such as *iNOS* and *COX-2*. In addition, it also suppresses the expression of pro-inflammatory cytokines, such as *TNF-α*, *IL-1β*, and *IL-6* [59]. To determine the underlying mechanism through which the lipid fractions of *A. japonicus* inhibit the nuclear translocation of NF-ĸB p65, we evaluated the protein levels of NF-κB p65 in cytosol and nucleus. Figure 4 shows that all kinds of lipid fractions suppressed the phosphorylation of NF-κB p65 in LPS-stimulated RAW264.7 cells. Furthermore, extracellular signal-regulated kinase (ERK), c-Jun N-terminal kinase/stress-activated protein kinase (JNK), and p38, were the MAPKs that played a major role in regulating pro-inflammatory cytokines [60,61]. All the lipid fractions of *A. japonicus* suppressed the LPS-stimulated phosphorylation of p38, ERK1/2, and JNK in a dose-dependent manner (Figure 4). In summary, these results indicate that NF-κB and MAPKs assisted the lipid fractions of *A. japonicus* in suppressing the production of NO and pro-inflammatory cytokines in activated macrophages.

## 4. Materials and Methods

### 4.1. Lipid Extraction and Separation

The total lipids of *Arctoscopus japonicus* eggs were extracted from a freeze-dried powder by using the Bligh and Dyer method (1959) [62]. Briefly, 4.5 g of the dried sample was homogenized with a mixture of chloroform/methanol (1:2 *v/v*). Then, we added 10 mL of chloroform and 10 mL of distilled water to the reaction mixture, and the blending process was continued for 30 s. In order to separate the two phases of the sample, we centrifuged it at 3000 rpm for 10 min. The chloroform phase was collected and dried under an evaporator. After evaporation, the samples were weighed to estimate the mass of lipids. Then, we dissolved the total lipids in hexane.

The total lipids were separated into different lipid fractions by performing silica gel column chromatography. The column was successively eluted with chloroform, acetone, and methyl alcohol to isolate neutral lipids, glycolipids, and phospholipids, respectively. After separation, the lipid fractions were dried and dissolved in dimethyl sulfoxide (DMSO) to achieve a final concentration of 20 mg/mL (*w/v*). Finally, the lipid fractions were stored at −20 °C for further analysis.

### 4.2. Fatty Acid Analysis

The Garces and Mancha method was used to extract fatty acids from the three lipid fractions of *A. japonicus*, namely, neutral lipids, glycolipids, and phospholipids [63]. The three lipid fractions were converted into fatty acid methyl esters (FAMEs) by performing a modified one-step lipid extraction method, which has been described in our previous study [64]. Then, the isolated FAMEs were analyzed by gas chromatography (GC)-flame ionization detection (FID) (Perkin Elmer, Waltham, MA, USA). The experiments were performed in pentaplicates (number of samples for each lipid fraction). The chromatographic peaks of FAMEs were identified by comparing their retention times with the internal standard C17:0 (Sigma-Aldrich, St. Louis, MO, USA).

### 4.3. The Measurement of Cell Proliferation

In this study, RAW264.7 cells (1 × 10^6^ cells/mL) were cultured for 24 h in RPMI-1640 medium, which was supplemented with 10% fetal bovine serum and 1% penicillin/streptomycin. Thereafter, different concentrations of the three lipid fractions (0.5%, 1.0%, 1.5%, and 2.0%) were treated and incubated for another 24 h. After incubation, the lipid fractions of *A. japonicus* were analyzed with the EZ-Cytox Cell Viability Assay Kit (DaeilLab Service, Seoul, Korea) to determine their cytotoxicity. The procedure has been described in a previous study conducted by Kim et al. [65]. Then, the absorbance of the lipid fractions was measured at 450 nm. The cell proliferation ability was estimated from the following formula:

$$\mathrm{Proliferation}\;\mathrm{ratio}\;\left(\%\right)=\frac{\mathrm{The}\;\mathrm{absorbance}\;\mathrm{of}\;\mathrm{the}\;\mathrm{sample}}{\mathrm{The}\;\mathrm{absorbance}\;\mathrm{of}\;\mathrm{the}\;\mathrm{control}}\times 100$$

### 4.4. The Measurement of Nitric Oxide (NO) Production

The cells were treated with different concentrations of each lipid fraction (0.5%, 1.0%, 1.5%, and 2.0%) for 1 h, and then they were treated with 1 μg/mL lipopolysaccharide (LPS) for 24 h. After stimulation, the NO concentration was estimated in the cultured medium by using Griess reagent (Promega, Madison, WI, USA) [66].

### 4.5. Qualitative Real-Time PCR

Total RNA was extracted from the cells by using the TRI reagent^®^ (Molecular Research Center, Cincinnati, OH, USA). Then, cDNA was synthesized with High-Capacity cDNA Reverse Transcription Kit (Applied Biosystems, Foster City, CA, USA), which was operated according to manufacturer’s instructions. To quantify the expression of immune genes, we used the following tools: SYBR^®^ Premix Ex Taq™ II (Takara Bio Inc., Shiga, Japan) and the QuantStudio™ 3 FlexReal-Time PCR System (ThermoFisher Scientific, Waltham, MA, USA). Table 2 presents the sequences of primers used in this analysis. The expression levels relative to the reference gene β-Actin were calculated by using the 2^−ΔΔCT^ method [67].

### 4.6. Western Blot Analysis

In this experiment, cells were pretreated with different concentrations of lipid fractions of *A. japonicus*. Then, these cells were stimulated with or without 1 μg/mL of LPS for 24 h. Proteins were extracted from these cells by using RIPA buffer (Tech & Innovation, Hebei, China). Protein concentrations were determined with Pierce™ BCA Protein Assay Kit (Thermo Scientific, Waltham, MA, USA). Equal amounts of protein (30 µg) were electrophoresed separately by performing sodium dodecyl sulfate-polyacrylamide gel electrophoresis (SDS-PAGE), and then they were transferred onto a polyvinylidene fluoride (PVDF) membrane. The membrane was incubated with primary antibodies that specifically identified the following transcription factors: p-NF-κB, p65, p-p38, p-JNK, and p-ERK1/2 (Cell Signaling Technology, Danvers, MA, USA), and α-tubulin (Abcam, Cambridge, UK). After washing the membrane, the bolts were incubated with Goat Anti-Rabbit IgG (H + L)-HRP (GenDEPOT, Katy, TX, USA) at 37 °C for 1 h. The bands were visualized with the following tools: Pierce^®^ ECL Plus Western Blotting Substrate (Thermo Scientific, Waltham, MA, USA), ChemiDoc XRS+ imaging system, and ImageLab software (Bio-Rad, Hercules, CA, USA).

### 4.7. Statistical Analysis

The results were expressed as mean ± standard deviation (SD). Significant differences were determined by performing a one-way analysis of variance (ANOVA) test with the help of SPSS software (Version 24, SPSS Inc, Chicago, IL, USA). The results were further analyzed by Duncan’s multiple-range test. The values at *p* < 0.05 were considered to be statistically significant.

## 5. Conclusions

The composition of fatty acids was determined in the three kinds of lipid fractions isolated from *A. japonicus* eggs. All the lipid fractions of *A. japonicus* contained a large amount of omega-3 PUFAs like EPA and DHA, which have several beneficial effects on human health. The present results indicate that lipid fractions of *A. japonicus* can effectively inhibit the production of NO in a dose-dependent manner. Moreover, these lipid fractions not only suppressed the expression of pro-inflammatory cytokines, such as *TNF-α*, *IL-6*, and *IL-1β* but also of pro-inflammatory mediators such as *iNOS* and *COX-2* in LPS-stimulated RAW264.7 cells. Furthermore, the lipid fractions of *A. japonicus* exhibited anti-inflammatory activity by suppressing the activity of NF-κB and MAPKs in LPS-stimulated macrophages. Therefore, the three lipid fractions of *A. japonicus*, namely neutral lipids, glycolipids, and phospholipids, could be potentially used as anti-inflammatory agents in the treatment of inflammatory diseases.

## Figures and Tables

**Table 1:** Fatty acid composition (wt.%) of fractionated lipids from *A. japonicus* eggs.

Fatty Acid	Neutral Lipids	Glycolipids	Phospholipids
Saturated fatty acid (SFA)			
16:0	33.17 ± 0.28 ^aA^	26.30 ± 0.27 ^aB^	19.01 ± 1.27 ^bC^
18:0	3.82 ± 0.05 ^fB^	6.23 ± 0.06 ^gA^	6.23 ± 0.42 ^eA^
Total SFAs	37.00 ± 0.31	32.53 ± 0.33	25.24 ± 1.68
Monounsaturated fatty acid (MUFA)			
16:1n7	9.874 ± 0.28 ^cA^	7.51 ± 0.05 ^fB^	-
18:1n9	25.94 ± 0.50 ^bA^	22.18 ± 0.23 ^bB^	15.83 ± 1.00 ^cC^
18:1n7	10.07 ± 0.14 ^cB^	8.98 ± 0.02 ^eC^	12.07 ± 0.73 ^dA^
20:1	1.05 ± 0.06 ^hA^	-	-
Total MUFAs	46.80 ± 0.79	38.68 ± 0.26	27.89 ± 1.73
Polyunsaturated fatty acid (PUFA)			
18:2n6 (LA)	1.14 ± 0.02 ^hA^	-	-
18:3n3 (ALA)	0.44 ± 0.02 ^iA^	-	-
20:3n3	1.99 ± 0.08 ^gC^	3.49 ± 0.06 ^hB^	6.21 ± 0.25 ^eA^
20:5n3 (EPA)	7.83 ± 0.35 ^dC^	12.07 ± 0.21 ^dB^	18.96 ± 1.27 ^bA^
22:6n3 (DHA)	4.80 ± 0.65 ^eC^	13.23 ± 0.33 ^cB^	21.70 ± 1.92 ^aA^
Total PUFAs	16.20 ± 0.70	28.79 ± 0.58	46.86 ± 3.40

Results are presented as means ± SD (*n* = 5). The letters (a–i) indicate significant differences (*p <* 0.05) between the amounts of fatty acids, which were obtained from the same *A. japonicus* lipid-fractions. The letters (A–C) indicate significant differences (*p* < 0.05) between the amounts of fatty acids, which were obtained from different *A. japonicus* lipid-fractions.

**Table 2:** The primers used in this study.

Target Gene		Sequence (from 5′ to 3′)
IL-1β	Forward Reverse	GGGCCTCAAAGGAAAGAATC TACCAGTTGGGGAACTCTGC
iNOS	Forward Reverse	TTCCAGAATCCCTGGACAAG TGGTCAAACTCTTGGGGTTC
IL-6	Forward Reverse	AGTTGCCTTCTTGGGACTGA CAGAATTGCCATTGCACAAC
COX-2	Forward Reverse	AGAAGGAAATGGCTGCAGAA GCTCGGCTTCCAGTATTGAG
TNF-α	Forward Reverse	ATGAGCACAGAAAGCATGATC TACAGGCTTGTCACTCGAATT
β-Actin	Forward Reverse	CCACAGCTGAGAGGAAATC AAGGAAGGCTGGAAAAGAGC
